# Role of Relational Ties in the Relationship between Thriving at Work and Innovative Work Behavior: An Empirical Study

**DOI:** 10.3390/ejihpe10010017

**Published:** 2019-11-19

**Authors:** Sidra Riaz, Yusen Xu, Shahid Hussain

**Affiliations:** 1 Faculty of Management and Economics, Dalian University of Technology, Dalian 116024, China; 2 Department of Mathematics, COMSATS University Islamabad, Attock Campus 43600, Pakistan; shahid_libra82@hotmail.com

**Keywords:** thriving at work, relational resources, strong ties, weak ties, heedful relating, innovative work behavior

## Abstract

Top management in organizations have begun to realize that innovative employees add to the competitive edge of a company which serves to maintain their position in intense market competition. For this purpose, management needs to seek new ways to combine the social environment and employees in the workplace in an inextricable manner that supports innovation. The purpose of this paper was to examine the role of thriving at work and its effects on an individual’s innovative behavior. Based on the socially embedded model of thriving, we aimed to assess the relevant related work on structured potential effects with relational ties (i.e., strong versus weak). Particularly, these ties affect the heedful relating differently. This study examined the antecedents of thriving at work and the innovative behavior among employees at a global investment company. Using partial least squares modeling on a sample of 412 observations (strong and weak ties), strong support was found for the theory-driven hypothesized relationships. The results contribute to a better understanding of the relational roles concerning recently emerging constructs of “thriving at work” and “positive organizational scholarship.” The implications and limitations of this study are further discussed.

## 1. Introduction

The increasing effects of international trade have generated concerns for local manufacturers, especially in developing countries. In order to compete with the tough competition created through the rising flow of imported goods, local manufacturers need to develop an innovative workforce and adopt new working strategies [1]. According to previous empirical research, employee engagement is not enough for organizations to survive, as there is a need to identify more factors that may help managers attain energetic and innovative employees. Therefore, Spreitzer et al. [2], in a socially embedded model, explained thriving as an organizational construct that is significantly associated with employees’ feeling of progress and momentum at work [3,4]. Thriving can be defined as a “psychological state in which individuals experience both a sense of vitality and learning at work” [2].

Recent research has focused on integrating thriving with performance, health, and wellbeing [5,6,7]; however, few studies have attempted to find the precursors of thriving at work [3,4]. Literature suggests that thriving at work is positively related to connectivity and trust [8], while the latter also states that individuals’ characteristics, such as regulatory focus, affect the sense of thriving [3]. Employees who experience more gratification regarding sense of thriving at work may feel that they are in a more advantageous position to reciprocate in terms of displaying innovative behavior. A rationale for this relationship can be attributed to the effect this has on an individual’s growth and cognition as well as the resulting behavior. Borrowing from Carmeli and Spreitzer [8], the first objective of this study is to examine the relationship between employees’ feelings of thriving at work and innovative behavior. The preceding arguments regarding thriving’s impact on the key determinants of heedful relating and innovative behavior suggest that effect of relational ties may vary as to how they heedfully relate.

Drawing on social cognitive theory [9], Spreitzer et al. [2] stated that more active and purposeful work provides the experience of psychological states that define thriving: vitality and learning. However, argentic work behaviors are the engine of thriving such as task focus, exploration, and heedful relating. Niessen et al. [10], in their diary study of thriving at work, explored the relationships of agentic work behaviors. Previous research demonstrated that most of the predicted relationships were between task focus and thriving [3,4,5] as proposed in the seminal work of Spreitzer et al. [2]. However, this study explains the role of heedful relating to influence individuals’ feelings of thriving and how it facilitates innovative behavior in comparison to Niessen et al.’s [10] findings. Niessen et al. [10] states that heedful relating, as per other agentic behavior (i.e., task focus and exploration), is less likely to predict thriving.

In addressing the above purposes, this study makes a major contribution to the field of thriving at work. First, it enriches the literature on thriving [2,5,11] by verifying the role of heedful relating as an important agentic work behavior to increase an individual’s feeling of thriving at work. Second, the empirical investigation extends the literature by explaining the role of relational resources more precisely. Third, by examining the role of heedful relating, this study elaborates that thriving at work impacts on individual behavior as more relational resources heedfully relate. Lastly, in view of the critical role of relational ties, we posit that heedful relating not only increases individuals’ feelings of thriving but also influences innovative behavior and increases organizational effectiveness. This study will also help to improve Pakistani manufacturers in their organizational practices

## 2. Literature Review and Conceptual Framework

### 2.1. Thriving at Work 

In recent years, thriving as a proliferating construct has received a great deal of scholars’ attention, and it is considered to be closely related to organizational outcomes such as better performance, employees’ self-development, and work engagement [3,7,12]. However, it still needs more empirical development to distinguish and demonstrate that it is as an organizational construct [11]. Thriving, based on (1) learning and (2) vitality creates the sense of improving at work and a feeling of increased energy [7], it also reflects the individual’s growth and progress at work [2,7,13]. In the initial works on thriving, Spreitzer et al. [2] described the distinguishing characteristics of thriving as subjective well-being, self-actualization, and flourishing. While thriving is acknowledged as a shared conceptual space with resilience, authors argued that the differences among these concepts are only particular circumstances. For example, “resilience refers to behavioral capacities that allow one to bounce back from untoward events, while the thriving focus on the positive psychological experience of increased learning and vitality to develop oneself and grow at work” [2] (p. 538). Furthermore, thriving can occur without any extreme and extenuating challenge, hence people can experience vitality and learn without adversity. Spreitzer et al. [2] (p. 539) argue that “if the right enabling conditions and resources are present, there is an increased likelihood that individuals will thrive, even under onerous conditions” [2] (p. 539). Based on these studies, the enabling conditions and contextual factors that can influence thriving at work needs to be further investigated. 

### 2.2. Thriving and Innovative Behavior 

Innovative work behavior (IWB) is based on new and useful ideas, process, or procedures that aim to achieve within a work role, group, or organization [14]. It includes problem detection or comes up with new ideas, solutions, and implementation. However, thriving serves as a gauge to monitor their own development and helps to improve the effectiveness and adaptability at work [2,3,12]. When individuals experience more thriving, they may be more likely to have the motivation to engage in innovative behavior [8,15,16] and go beyond their regular role as well as responsibilities. Sonenshein et al. [17] noted that thriving is distinct from intrinsic motivation, because of the vitality it derives as a desire to perform such behavior on effective enjoyment way and learning impetus of individual for goal achievement and personal recognition.

According to previous studies, an individual’s innovative behavior is related to learning and development at work which enables the employee to recognize problems and implement improvements [3,8]. In this process, an employee needs to learn the required expertise to understand the process, distinguish the problems, and find a creative solution [8]. Second, employees must be energetic to devote to implementing the new process, whereas psychological and social resources facilitate thriving and allow individuals’ to be more innovative [3]. Previous empirical work supports the link between thriving and innovative behavior [8,12,18,19]. Thus, we hypothesize that

**H_1_:**  Thriving will be positively associated with innovative work behavior.

### 2.3. Heedful Relating and Thriving 

Heedful relating can be defined as a social process of individuals through ongoing activities of representing, contributing, and subordinating [20]. In organizations, employees work in groups or as a team member, such collaborative and interdependent work play the role of a social force to achieve common goals, [15,21]. The concept of heedful relating has been discussed in terms of social force which works with purposeful, careful, and conscientious interpersonal interaction to provide workers with opportunities to learn from others job through the generation of new ideas for growth [22,23]. Moreover, Heaphy and Dutton [24] demonstrate that heedful relating expands the individual sense of understanding others’ job which facilitates people to take the risk and enhance their learning and growth. Positive organizational scholarship research reveals that work relationships ingrained in heedful relating can energize individuals [2,4,22].

Furthermore, heedful relating is also related to learning component of thriving, as Paterson et al. [4] argue that employee’s interaction at work is the way to learn new knowledge and refine their skills. As social learning theory suggests, employees learn from others job, look after each other at the workplace by giving them a chance to learn new work methods [9]. Positive interaction of employees with co-workers provides the chance to learn new knowledge. Literature gives the support that employee learning at work depends on heedful interaction—through observing others’ work, cooperatively responding in work, and talking about work [4,23,25,26]. Additionally, social connections are the source of knowledge for each other which depends on their heedfulness (see [8]).

In thriving research, Carmeli and Spreitzer [8] suggested that trust and connectivity are the primary factors to increase thriving (while connectivity relates to heedful relating as consistent construct). However, the difference is heedful relating not only creates social support but also tries to fit with others job, which is relatively high to the accomplishment of common goals. Individuals involved in the execution of all processes may feel more sense of learning [9], in line with the learning dimension of thriving. Moreover, when individuals work in an interrelated and cooperative manner, they feel more social support that can enhance the sense of vitality and zest, such as the second dimension of thriving [27]. Based on these arguments we predict that heedful relating may promote the sense of learning from coworker’s jobs, enabling them to learn new approaches to align their work with others in order to accomplish a common goal more effectively. Hence, heedful related work relationship can promote individuals’ sense of learning and acquiring new skills. Thus, we suggest that:

**H_2_:**  Heedful relating will be positively associated with thriving.

### 2.4. Heedful Relating, Thriving and Innovative Behavior 

It can also be expected that heedful relating will also have a positive effect on innovative behavior [11]. More specifically, Abid et al. [22] and Bijlsma-Frankema et al. [28] reported that task programmability work structures support individuals’ to do their part of the job more efficiently and help them to indicate the flaws. Moreover, social support encourages to implement new ideas. However, Kanter [29] found that individual assessment to fulfill the task in a participative, collaborative, and communicative way help the individuals’ to focus on given task and it may become more convenient to him/her to understand the whole process and efficiently configure the creative ideas. Many studies support the argument that positive relations among supervisor and subordinates help to alleviate stress at the workplace, provide social support and openness, as well as enhance creativity [11,30,31]. All of these findings are consistent with the argument that heedful relating should also be related with innovative work behavior.

Furthermore, literature evidence suggested the direct link between heedful relating and thriving at work [13]. We therefore propose that thriving will further mediate the relationship between heedful relating and innovative work behavior. The work connectivity provides the opportunity to learn from others’ work—as the first dimension of thriving whereas, relationships at work provide support, connectivity, and openness, as well as individuals experience more vitally at work—as the second dimension of thriving. Individuals are more likely to feel free to share their ideas and information with each other when there is more connectivity. Thus we hypothesized that

**H_3_:**  Thriving will mediate the relationship between heedful relating and innovative behavior.

### 2.5. Thriving and Relational Ties 

According to Provan et al. [32] many terms have used in literature for relational ties that characteristically share the common theme. These relationships are based on reciprocity, connectedness, and collective action. However, “tie” is considered as the basic building block to explain particular interaction. Halldorsson et al. [33] affirmed that inter-organizational cooperative relations exert a major influence on controlling and coordinating task.

Moreover, social network theory considered the web of social ties as capable of conveying, representing, influencing perceptions, expectations, and ideas among each other [8]. These relationships emerge in organizations and facilitate the exchange of information and energy. As the dimension of thriving, we may expect that the exchange of relational resources among different departments can lead to more thrive at work. Previous studies considered “trust” as the main component (e.g., [8]), while more interactive work can also be a cause to feel more progress and energy at work.

An individual’s within-group relations with peers and leaders are considered as strong ties which is developed and maintained by an individual on a reciprocity basis, more emotional closeness, and frequent interactions [34]. Whereas, weak ties involve few interactions and comparatively low emotional closeness. However, [35] provides the pockets of non-redundant information from a diverse perspective and increases individuals’ learning. Previous research has found that weak ties can facilitate the process of knowledge and energy exchange [5,19,36,37].

Although literature gives the support that relational ties are the source of inspiration, support, and learning [37,38], there is considerable controversy on the effects of relational ties on organizational outcomes. An empirical investigation needed to understand such a path of combinatory process of self-development and energy toward innovation affects the organizational progress [38]. Carmeli and Spreitzer [8] suggested that workplace relational resources can play a crucial role in learning through their experience and effect on co-workers’ vitality and encouragement. So we hypothesized that:

**H_4a_:**  Strong ties will be positively related to thriving.

**H_4b_:**  Weak ties will be positively related to thriving.

Furthermore, Spreitzer et al. [2] suggested that the work context including how information exchanged influences the extent to which individuals engage in active and purposeful actions. However, Jiang [18] also considered heedful relating as an important mediator for individuals’ relational resources.

Paterson, et al. [4] noted that heedful relating involves the generation of parallel work environments that connects with others. Literature indicates such agentic behavior as a primary mediator that largely shaped individual behavior [23]. Specifically, social resources effectivity depends on connections and interaction, hence it is essential to determine the work structure that supports connectivity and enhances thriving at work [25,26,28,39]. Thus we hypothesize that,

**H_5a_:**  Heedful relating will mediate the relationship between strong ties and an employee’s sense of thriving.

**H_5b_:**  Heedful relating will mediate the relationship between weak ties and an employee’s sense of thriving.

## 3. Method 

### 3.1. Sample and Procedure 

Data for this current study was collected from the employees of textile manufacturers in Pakistan. Preliminary interviews revealed that respondents were middle-level professionals and need to coordinate with colleagues of their department as well as with other departments (i.e., production, marketing, sales). The human resource department provided a list of 300 employees and half of them were willing to participate in the present research. While, before the participation, they gave their informed consent for inclusion in this study. The study was conducted in accordance with the Declaration of Helsinki, whereas protocol was approved by the Ethics Committee. After subtracting incomplete surveys a total of 118 respondents completed the entire survey (response rate = 39.3%). 

The final respondents reported on four relationships at work, resulting in 472 total sample of observations. Among 81% respondents who were males, 44% were bachelor degree holders while the remaining had some professional degree. Average working experience of each respondent in this company was 5.2 years, whereas the median age was evidenced to be in the early 40s. Also, non-respondent demographic information which included only gender consisted of 82% men. We found non-significant results of interaction effects between the predictor variable and dummy variable and these results were the same for all data, so we pooled the data for analysis.

### 3.2. Measures 

#### 3.2.1. Innovative Work Behavior

To measure the innovative work behavior we adopted the scale of Scott and Bruce [14] which consist of six-items. Employees were asked to report accordingly to the extent to which they engage and display innovative behavior at work. Responses were made on a five-point Likert scale ranging from 1 = “strongly disagree” to 5 = “strongly agree” scale item included (i.e., “I generate creative ideas at work” and “I always try to seek out new techniques and technology to improve the process or product ideas at work”α = 0.903).

#### 3.2.2. Thriving at Work

We used 10 items to measure the learning and vitality with scale by Porath et al. [7] ranging from “1 = strongly disagree” to”5 = strongly agree.” To measure learning, five items were used as sample items (“At work I find myself learning often”) while five items were used to measure the feeling of vitality or energy at work sample item: (“I feel alive at work”) α = 0.940.

#### 3.2.3. Heedful Relating

To measure heedful relating we used the scale developed by Bijlsma-Frankema et al. [28]. Five-point scales were used ranging from 1 = not really to 5 = always. For example “My job demands to coordinate my action with my co-workers contribute to our success” “I fully participate in my work with other α = 0.922.

#### 3.2.4. Relational Ties

To assess the data to strong ties and weak ties following the Petróczi et al. [40] study, we also arranged the groups (i.e., N1, N2 where N is respective employee department). We asked respondents to examine the list of their colleagues and other department employees’ which he/she needs to coordinate frequently.

### 3.3. Control Variables 

To reduce the likelihood of work expertise that may account for variance in innovative behavior [41,42] we employed organizational tenure as a control variable together with age, education, and gender. Previous research suggests these factors can affect the innovative behavior [8,15]. However, gender as a controlled variable is considered for non-respondents.

## 4. Results 

This study used the partial least square (PLS) technique for data analysis by using SmartPLS 3.0 software to estimate the research model. The main objective for using the PLS approach is that the research model of the study contains second-order reflective-reflective constructs of which the PLS methods is able to accommodate the latent constructs to be modeled either as reflective or formative constructs and has a lower restriction regarding sample size [43,44,45]. A listwise deletion of missing values reduces our sample from 472 to 412 (and from 118 to 103 respondents). By following Petróczi et al. [40], we included 102 dummy variables to represent 103 respondents to account for any non-independence, because each respondent reported on four strong and weak ties. The reliability, validity, and assessment measurement model of first-order reflective indicators were examined in the first stage while testing of structural model was established in the second stage. The correlation between variables along with means and standard deviations are presented in Table 1. All correlations are significant at α=0.01.

### 4.1. Evaluation of Measurement Model 

All the first-order variables were measured reflectively. Table 2 represents the results of each measurement scale (average variance extracted (AVE), composite reliability (CR), and Cronbach’s alpha) and discriminant validity (Fornell-Larcker Criterion). All reflective loadings ranged from 0.721 to 0.862 and significant at 0.01 level. All values of CR and Cronbach’s alpha are greater than 0.70, indicating high levels of internal consistency reliability. The next measure is convergent validity, which defines as the model’s ability to explain the indicator’s variance. Fornell and Larcker [46] suggested AVE can provide evidence for convergent validity and [47] suggest the threshold for AVE is 0.5. In this study, the AVE, ranges from 0.619 to 0.681 well above the required minimum level of 0.50 [47], indicating a high level of convergent validity. Finally, considering the discriminant validity of this model assessed by Fornell and Larcker [46], Table 2 shows that the variables fulfill the Fornell-Larcker criterion and discriminant validity for this research.

This study follows Hair Jr, et al. [48]’s five-step approach to measuring structural model: (1) Collinearity assessment among the constructs, (2) structural model path coefficients, (3) coefficient of determination ($R^{2}$ value), (4) effect size $f^{2},$ and (5) predictive relevance $Q^{2}$ and blindfolding. The details of each step appear below.

First, this study examines each set of predictors in the structural model for possible collinearity. Variance inflation factor (VIF) indicates the problem of collinearity if its value is 5 or above [48]. All VIF values are lower than five, suggesting that there is no indication of collinearity between each set of predictor variables. 

**Figure 1:**
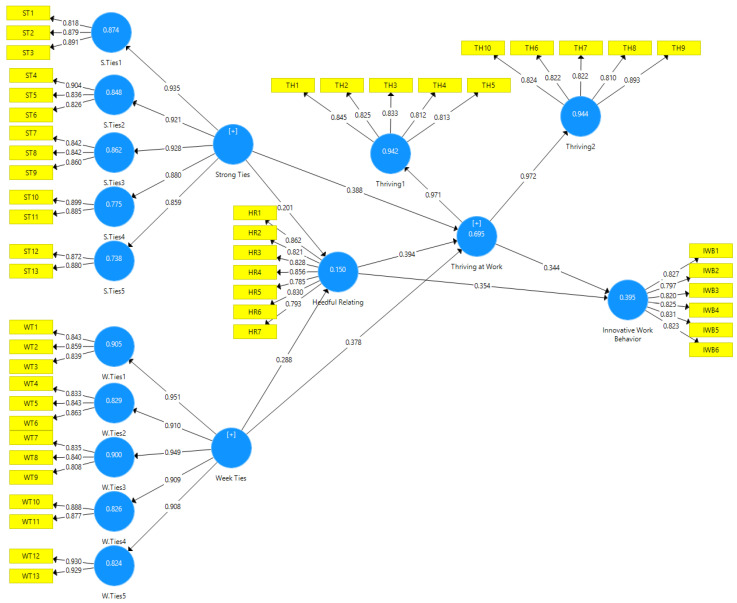
Measurement model with factor loadings.

Second, this study uses a 3000 bootstrapping samples and each sample containing the same number of observations as the original sample (i.e., 412 bootstrap cases) to generate standard errors and *t*-values [43,48,49]. The study assesses estimated path relationships among the latent variables in the model through the sign and magnitude of the path coefficients. The results are presented in both tabular and graphical forms. Table 3 summarizes the results shown in Figure 1.

Falk and Miller [50] recommended the value of $R^{2}$ should be at least 10% for endogenous constructs. The $R^{2}$ values for this study are above adequate level as the value of $R^{2}$ for heedful relating is rather weak (0.159) by following the rule of thumb, whereas the values for thriving at work is high (0.695) and moderate for innovative work behavior (0.395).

Fourth, this study also calculates the $f^{2}$ and $q^{2}$ effect sizes. The suggested values of $f^{2}$ and $q^{2}$ are 0.02, 0.15, and 0.35 for small, medium, and large effects, respectively [49]. Table 3 also represents the values of $f^{2}$ and $q^{2}.$

**Figure 2:**
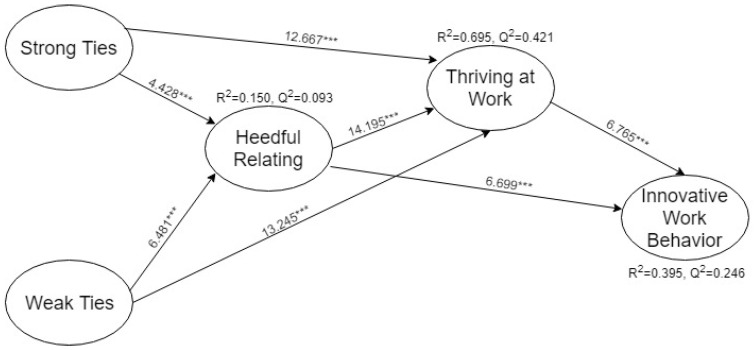
Assessment of structural model.

Finally, the values of $Q^{2}$ is calculated by using the procedure of blindfolding. All values of $Q^{2}$ for this study are above zero, providing support for the model’s predictive relevance regarding the reflective endogenous latent variables. The values of $Q^{2}$ are presented in Figure 2 along with the values of $R^{2}.$

From Table 3, the relationship between thriving at work and innovative work behavior is highly significant (β=0.3543, p<0.000) supporting $H_{1}.$ Similarly, $H_{2}$ is also supported by the highly significant relation between heedful relating and thriving at work (β=0.3935, p<0.000). In addition, the relationship between strong ties and weak ties with thriving is also positive and significant as (β=0.3881, p<0.000) and (β=0.3781, p<0.000) supporting $H_{4a}$ and $H_{4b},$ respectively.

### 4.2. Mediation Assessment 

This study analyzes the significance of three mediation effects by adopting the bootstrapping method [48,51]. The requirement for the mediation is that the indirect effect has to be significant and 95% bootstrapped confidence interval should not contain the value zero. To test our hypothesis 3 that thriving at work will mediate the relationship of heedful relating and innovative work behavior, we checked both direct and indirect effects. The direct effect of heedful relating and innovative work behavior is significant with (β=0.569, p<0.000). After introducing mediated variable thriving at work in the model the estimate of heedful relating and innovative work behavior is (β=0.354, p<0.000) still significant but reduced. So, we can conclude that the thriving at work is mediates the relationship between heedful relating and innovative work behavior. The bootstrapping analysis showed that the indirect effect was significant with (β=0.1355, SE=0.0226, t=5.997). Also, as indicated by Preacher and Hayes [51], the 95% bootstrapped confidence interval does not straddle a value zero in between which also indicates the existence of mediation and supports $H_{3}.$ Similarly, from Table 4, the results fulfill all the necessary conditions for the assessment of heedful relating as a mediator between strong ties and thriving at work as well as weak ties and thriving at work using bootstrapping procedure. So, our hypotheses $H_{5a}$ and $H_{5b}$ are also true.

## 5. Discussion 

The findings of the current study indicate that thriving at work enhances innovative behavior. The results of the current study provide the empirical support for the relationship proposed by Spreitzer et al. [2] and also consistent with previous studies (e.g., [3,8]) which state that individuals feeling of thriving at work affect their behavior. One reason for such a significant relationship between thriving at work and innovative behavior may be that when an individual works more enthusiastically, he or she feels more energetic to display innovative behavior.

As an agentic work behavior, heedful relating also is positively related to thriving at work while results also show that an individual’s subordination at work provides more opportunities to learn. Results are aligned with previous study findings of [4,22]. In a few studies, it has been found less effective as an agentic variable related to thriving at work (i.e., [10]). The reason for such a significant relationship between heedful related work and thriving may be that when individuals work with more consciously, they feel more involved in their work and learn.

The relationship between heedful relating and innovative behavior with the role of thriving as mediator indicate the support for the aforesaid proposition of Carmeli and Spreitzer [8]. Also, the results of the current study are aligned with the findings of the previous studies that thriving at work enhances individuals’ innovative behavior [3,4,8]. Current study explained the valuable effects of heedful related work and thus supports the argument that an agentic behavior can also positively affect innovative behavior.

The results of the current study support the findings of [21,52], indicating that relational ties at work positively affect individual learning. Peers and leaders provide adverse information and zest [37], while Gerbasi et al. [5]’s study indicates relational ties as the source to encourage or de-energizing the individual at work. Based on previous study findings, this current study explains such positive relation as the effect of relational ties which increases as individuals’ feeling of thriving work heedfully. The underlying reason for heedful relating is due to an increase of mindfulness [8,53] which means that employees do interrelated work to achieve organizational goals. That provides the chance to learn more from others and boost their energy. Moreover, the results illustrate that individual’s thriving at work become more enhanced and highly effective when weak ties relate heedfully. The reason is that weak ties have a less emotional attachment and interaction; the way to learn from weak ties can be heedful related work. It is pertinent to note here that any previous study has not yet examined this relationship. Therefore, this study provides an empirical support to a proposed relationship that weak ties can be more benefited if heedfully relate.

### 5.1. Theoretical Implications 

The study findings have several theoretical implications. First, the most significant contribution of current research could be the more determined focus of relational resources in the socially embedded model of thriving [2]. Specifically, this study incorporates the exploratory mechanism to investigate the different relational resources effects on thriving, whereas the results are significantly higher when heedful relating as an agentic work behavior effectively contribute to this relation. Based on the literature, this present study attempts to bring collaborative work effect on employee’s innovative behavior. In doing so, this study responds to recent calls by Wallace et al. [3] for research to examine how thriving as mediator relates with relational resources and innovative behavior at work and role of heedful relating as an important agentic behavior. Furthermore, this study contributes to thriving literature by using heedful relating as an important agentic behavior of thriving. Thus, the meaningful implication of the study is to empirically examine the effects of thriving at work as a mediator, as well as to understand the complex nature of organization and individual that can operate together more effectively to thrive and innovate. Heaphy and Dutton [24]’s study shows that work relationships are important factors in individuals’ energy at work. Our results extend the knowledge that more heedful relating heightened the sense of vitality.

Second, our results have implications for the organizations for collaborative focused strategies that enhance thriving and subsequently innovation. Even though cumulative research argues that relational resources are important predictors of thriving [54] but not found any research that has devoted to examining how this effect occurs. The present research addressed this resource by showing that different relational ties with heedful relating can affect the individuals’ thriving unlikely. Conversely, study findings suggest that those employees who focus on the organizational goals and generate social force can experience more thriving and exhibit innovative behavior [3,4,8,53]. Finally, study findings highlight the important role of heedful relating as agentic work behavior that promotes an individual’s feeling of thriving effectively contrary to the results of [10]. Moreover, it enhances the importance of weak ties that can be more effective when employees perform mutual task heedfully. However, individual feel more thriving and exhibit innovative behavior when heedfully relate with relational resources to accomplished organizational goals.

In summary, by supporting the complex and multi-relationships model, the present study has extended previous theory and research on the process of innovation [8,29] based on social cognitive theory [9]. In doing so, it provides a more comprehensive understanding of employee relations and collaborative work that affect the innovative behavior and the role of thriving as a major explanatory mechanism. Moreover, findings were obtained through the time-lagged design across three points in time, which has not focused on prior work. The methodological strength of the study, coupled with the use of multipath analysis estimated all relationships one by one, which attests to the robustness of the study. Moreover, the findings and their relevance helps to understand the individuals’ innovative behavior with enabling and motivating contextual factors.

### 5.2. Practical Implications 

The results bring along some valuable suggestions that if organizations adopt heedful employee-focused strategies can benefit through more innovative behavior. Present research based on the textile industry needs to align the line of production (i.e., production, marketing, sales, and customer demands). To fulfill these purposes innovatively it is important that employees work carefully, consistently, critically, attentively, conscientiously, pertinaciously, purposefully, and vigilantly [53]. However, these abilities mean employees work heedfully not as habitual performance can also increase innovation. Thus, actions based on heed can improve the employees’ ability to work together. Further, giving care and attention to co-workers’ new ideas can enhance team effectiveness. When employees feel like a part of a system based on a shared mental model characterized by a mutual understanding of themselves and the environment when works heedfully. If organizations train the employee not only about their task but also the inter-link of their task with others can help them to implement new ideas. For example when individuals know their task importance in the whole system then he/she could be more innovative at his/her task to increase performance and benefit through diverse information of relational resources. While heedful relating is a more effective way to learn more from these resources more specifically from weak ties. As for the weak ties, individual has less closeness, less interaction but can get diverse ideas. In a high-reliability context, interrelating work activities provide more opportunities to learn from each other and enhance innovative behavior. The manager can also adopt the heedful strategy to transfer knowledge toward new employees to fill the hole that generates whenever any experienced employee quit the organization. The current study induces that heedful related relational resources are important for innovative behavior as much as for thriving at work.

### 5.3. Limitations and Future Recommendations 

Although this study makes an important contribution to understanding the role of individual thriving in the innovation process in the relational perspective, also have limitations. First, the sample was drawn from a few organizations, thus these findings may not generalize to other organizations. As previous research noted that more social ties provide more diverse information [55]. Future research can examine the role of other relational resources (such as organizational out-group) is thriving and more explicitly incorporate with innovation. This study will encourage researchers to further explore the constructs of mediating mechanism and contribute into positive organizational scholarship.

Although the current study used a rigorous time-lagged design consisting of three months, following the prior studies of [3,8] but cannot be considered as longitudinal. To examine more precise results of weak ties, the present study encourages the researchers to build on our model in more longitudinal design to examine the more strong effects of the weak ties reciprocal effect of thriving and innovation which proposed by Spreitzer et al. [2].

Third, although we examined the important relational resources, also there are other personal resources (e.g., family, friends, prior job colleagues) that could account for individual innovative behavior [34]. Moreover, we just controlled the gender variable for non-respondent, but for other variables like education, age, the experience might have a greater effect [4,7]. Fourth, although we theorized heedful relating as an important antecedent of thriving but with un-effective heedful relating the group performance can be adverse [20]. Future studies should also consider these elements. 

## 6. Conclusions 

This study investigated the process that how relational resources can be more effective with the mediating role of heedful relating and thriving at work to increase innovative behavior. Moreover, it precisely examined the role of relational ties (strong ties and weak ties) in the promotion of thriving. In addition, heedful relating as an important agentic behavior increases the feeling of workplace thriving and innovation subsequently. Furthermore, weak ties can be more effective when heedfully relate. Moreover, such an energetic workforce exhibit more innovative behavior. These results theoretically and empirically extended the thriving model based on cognitive theory to shed light on developing and increasing the effectivity of relational resources. The study findings can help organizations to develop a more effective working design to increase employee innovative behavior.

## Figures and Tables

**Table 1:** Descriptive and correlation matrix.

	M	SD	(1)	(2)	(3)	(4)	(5)
Innovative Work Behavior (1)	2.756	0.939	1.000				
Thriving at Work (2)	3.071	0.938	0.554 **	1.000			
Heedful Relating (3)	3.142	0.875	0.555 **	0.615 **	1.000		
Strong Ties (4)	3.208	0.824	0.325 **	0.571 **	0.262 **	1.000	
Weak Ties (5)	3.159	0.832	0.319 **	0.594 **	0.325 **	0.221 **	1.000

Note. N = 412, M = means, SD = standard deviation, * *p* < 0.05 (two-tailed), ** *p* < 0.01 (two-tailed).

**Table 2:** Measurement model and discriminant validity (Fornell-Larcker Criterion).

	AVE	CR	Cronbach’s Alpha	(1)	(2)	(3)	(4)	(5)
Heedful Relating (1)	0.650	0.949	0.940	0.826				
Innovative Work Behavior (2)	0.674	0.925	0.903	0.568	0.821			
Strong Ties (3)	0.619	0.955	0.948	0.266	0.330	0.787		
Thriving at Work (4)	0.650	0.949	0.940	0.623	0.564	0.578	0.806	
Weak Ties (5)	0.637	0.958	0.952	0.334	0.327	0.226	0.597	0.798

Note. N = 412, AVE = average variance extracted, CR = composite reliability.

**Table 3:** Significant testing results of the structural model path coefficients.

Path	Path Coefficients	Mean	SD	tValues	pValues	$\mathrm{Effect}\;\mathrm{Size}\;(f^{2})$	$\mathrm{Effect}\;\mathrm{Size}\;(q^{2})$	CI (2.5–97.5)%
HR -> IWB	0.3543	0.3556	0.0529	6.6992	0.0000	0.1273	0.1158	(0.2513, 0.4571)
HR -> Thriving	0.3935	0.3945	0.0277	14.1953	0.0000	0.4328	0.3981	(0.3397, 0.4486)
S.Ties -> HR	0.2007	0.2029	0.0453	4.4282	0.0000	0.0447	0.0371	(0.1138, 0.2890)
S.Ties -> Thriving	0.3881	0.3875	0.0306	12.6666	0.0000	0.4492	0.4074	(0.3256, 0.4452)
Thriving -> IWB	0.3436	0.3434	0.0508	6.7655	0.0000	0.1190	0.1029	(0.2429, 0.4407)
W.Ties -> HR	0.2883	0.2889	0.0445	6.4814	0.0000	0.0917	0.0870	(0.2021, 0.3732)
W.Ties -> Thriving	0.3781	0.3776	0.0285	13.2447	0.0000	0.4066	0.3765	(0.3218, 0.4345)

**Table 4:** Results of direct and indirect relation by bootstrapping approach.

Hypothesis	Procedure	Path	Path. Coef.	Indirect Effect	STDEV	Total Effect	95% Boot Strapped CI	t Values	pValues	Decision
HR -> Thriving -> IWB	Step1: Direct effect (without mediator)	HR -> IWB	0.569	n/a				16.662	0.000	Accepted
	Step2: Indirect effect (with mediator)	HR -> IWB	0.354	n/a		0.7045	(0.0912, 0.1798)	5.9970	0.000	
		HR -> Thriv	0.394	0.1355	0.0226					
		Thriv -> IWB	0.344							
S.Ties -> HR -> Thriving	Step1: Direct effect (without mediator)	S.Ties -> Thriv	0.467	n/a				13.789	0.000	Accepted
	Step2: Indirect effect (with mediator)	S.Ties -> Thriv	0.388	n/a		0.5462	(0.0418, 0.1166)	4.1460	0.000	
		S.Ties -> HR	0.201	0.0792	0.0191					
		HR -> Thriv	0.394							
W.Ties -> HR -> Thriving	Step1: Direct effect (without mediator)	W.Ties -> Thriv	0.491	n/a				15.072	0.000	Accepted
	Step2: Indirect effect (with mediator)	W.Ties -> Thriv	0.378	n/a		0.6045	(0.0754, 0.1515)	5.8470	0.000	
		W.Ties -> HR	0.288	0.1135	0.0194					
		HR -> Thriv	0.394							

## References

[1] Imran M.K., Ilyas M., Aslam U., Fatima T. (2018). Knowledge processes and firm performance: The mediating effect of employee creativity. J. Organ. Chang. Manag..

[2] Spreitzer G., Sutcliffe K., Dutton J., Sonenshein S., Grant A.M. (2005). A socially embedded model of thriving at work. Organ. Sci..

[3] Wallace J.C., Butts M.M., Johnson P.D., Stevens F.G., Smith M.B. (2016). A multilevel model of employee innovation: Understanding the effects of regulatory focus, thriving, and employee involvement climate. J. Manag..

[4] Paterson T.A., Luthans F., Jeung W. (2014). Thriving at work: Impact of psychological capital and supervisor support. J. Organ. Behav..

[5] Gerbasi A., Porath C.L., Parker A., Spreitzer G., Cross R. (2015). Destructive de-energizing relationships: How thriving buffers their effect on performance. J. Appl. Psychol..

[6] Hennekam S. (2017). Thriving of older workers. Pers. Rev..

[7] Porath C., Spreitzer G., Gibson C., Garnett F.G. (2012). Thriving at work: Toward its measurement, construct validation, and theoretical refinement. J. Organ. Behav..

[8] Carmeli A., Spreitzer G.M. (2009). Trust, connectivity, and thriving: Implications for innovative behaviors at work. J. Creat. Behav..

[9] Bandura A. (1977). Self-efficacy: Toward a unifying theory of behavioral change. Psychol. Rev..

[10] Niessen C., Sonnentag S., Sach F. (2012). Thriving at work—A diary study. J. Organ. Behav..

[11] Carmeli A., Russo M. (2016). The power of micro-moves in cultivating regardful relationships: Implications for work–home enrichment and thriving. Hum. Resour. Manag. Rev..

[12] Riaz S., Xu Y., Hussain S. (2018). Understanding employee innovative behavior and thriving at work: A Chinese perspective. Adm. Sci..

[13] Spreitzer G.M., Sutcliffe K.M., Nelson D.L., Cooper C.L. (2007). Thriving in organizations. Positive Organizational Behavior.

[14] Scott S.G., Bruce R.A. (1994). Determinants of innovative behavior: A path model of individual innovation in the workplace. Acad. Manag. J..

[15] Dutton J.E. (2003). Energize Your Workplace: How to Create and Sustain High-Quality Connections at Work.

[16] Quinn R.W., Dutton J.E. (2005). Coordination as energy-in-conversation. Acad. Manag. Rev..

[17] Sonenshein S., Dutton J., Grant A., Spreitzer G., Sutcliffe K. (2006). Narrating of Growth at Work: Rationalist and Socio-Emotionalist and Logics of Development.

[18] Jiang Z. (2017). Proactive personality and career adaptability: The role of thriving at work. J. Vocat. Behav..

[19] Pallotti F., Lomi A., Mascia D. (2013). From network ties to network structures: Exponential Random Graph Models of interorganizational relations. Qual. Quant..

[20] Weick K.E., Cameron K.S., Dutton J.E., Quinn R.E. (2003). Positive organizing and organizational tragedy. Positive Organizational Scholarship: Foundations of a New Discipline.

[21] Dutton J.E., Heaphy E.D. (2003). The power of high-quality connections. Posit. Organ. Scholarsh. Found. A New Discip..

[22] Abid G., Zahra I., Ahmed A. (2016). Promoting thriving at work and waning turnover intention: A relational perspective. Future Bus. J..

[23] Druskat V.U., Pescosolido A.T. (2002). The content of effective teamwork mental models in self-managing teams: Ownership, learning and heedful interrelating. Hum. Relat..

[24] Heaphy E.D., Dutton J.E. (2006). Embodying social interactions: Integrating physiology into the study of positive connections and relationships at work. Acad. Manag. Rev..

[25] Crant J.M. (2000). Proactive behavior in organizations. J. Manag..

[26] Parker S.K., Ohly S. (2008). Designing motivating jobs. Work Motivation. Past Present Future.

[27] Brown S.L., Nesse R.M., Vinokur A.D., Smith D.M. (2003). Providing social support may be more beneficial than receiving it: Results from a prospective study of mortality. Psychol. Sci..

[28] Bijlsma-Frankema K., de Jong B., van de Bunt G. (2008). Heed, a missing link between trust, monitoring and performance in knowledge intensive teams. Int. J. Hum. Resour. Manag..

[29] Kanter R.M. (1988). Three tiers for innovation research. Commun. Res..

[30] Korzilius H., Bücker J.J., Beerlage S. (2017). Multiculturalism and innovative work behavior: The mediating role of cultural intelligence. Int. J. Intercult. Relat..

[31] Albrecht T.L., Hall B.J. (1991). Facilitating talk about new ideas: The role of personal relationships in organizational innovation. Commun. Monogr..

[32] Provan K.G., Fish A., Sydow J. (2007). Interorganizational networks at the network level: A review of the empirical literature on whole networks. J. Manag..

[33] Halldorsson A., Kotzab H., Mikkola J.H., Skjøtt-Larsen T. (2007). Complementary theories to supply chain management. Supply Chain Manag. Int. J..

[34] Perry-Smith J.E., Shalley C.E. (2003). The social side of creativity: A static and dynamic social network perspective. Acad. Manag. Rev..

[35] Baer M. (2010). The strength-of-weak-ties perspective on creativity: A comprehensive examination and extension. J. Appl. Psychol..

[36] Mumford M.D., Gustafson S.B. (1988). Creativity syndrome: Integration, application, and innovation. Psychol. Bull..

[37] Wang X.H.F., Fang Y., Qureshi I., Janssen O. (2015). Understanding employee innovative behavior: Integrating the social network and leader–member exchange perspectives. J. Organ. Behav..

[38] Kijkuit B., van den Ende J. (2010). With a little help from our colleagues: A longitudinal study of social networks for innovation. Organ. Stud..

[39] Bakker A.B., Tims M., Derks D. (2012). Proactive personality and job performance: The role of job crafting and work engagement. Hum. Relat..

[40] Petróczi A., Nepusz T., Bazsó F. (2007). Measuring tie-strength in virtual social networks. Connections.

[41] Oldham G.R., Cummings A. (1996). Employee creativity: Personal and contextual factors at work. Acad. Manag. J..

[42] Tierney P., Farmer S.M. (2004). The Pygmalion process and employee creativity. J. Manag..

[43] Chin W.W., Marcolin B.L., Newsted P.R. (2003). A partial least squares latent variable modeling approach for measuring interaction effects: Results from a Monte Carlo simulation study and an electronic-mail emotion/adoption study. Inf. Syst. Res..

[44] Lee C.-S., Chen Y.-C., Tsui P.-L., Yu T.-H. (2014). Examining the relations between open innovation climate and job satisfaction with a PLS path model. Qual. Quant..

[45] Terzi S., Trezzini A., Moroni L. (2014). A PLS path model to investigate the relations between institutions and human development. Qual. Quant..

[46] Fornell C., Larcker D.F. (1981). Evaluating structural equation models with unobservable variables and measurement error. J. Mark. Res..

[47] Bagozzi R.P., Yi Y. (1988). On the evaluation of structural equation models. J. Acad. Mark. Sci..

[48] Hair J.F., Hult G.T.M., Ringle C., Sarstedt M. (2016). A Primer on Partial Least Squares Structural Equation Modeling (PLS-SEM).

[49] Chin W.W. (1998). The partial least squares approach to structural equation modeling. Mod. Methods Bus. Res..

[50] Falk R.F., Miller N.B. (1992). A Primer for Soft Modeling.

[51] Preacher K.J., Hayes A.F. (2008). Asymptotic and resampling strategies for assessing and comparing indirect effects in multiple mediator models. Behav. Res. Methods.

[52] Baker W., Cross R., Wooten M., Cameron K., Dutton J., Quinn R. (2003). Positive organizational network analysis and energizing relationships. Positive Organizational Scholarship. Foundations in a New Discipline.

[53] Weick K.E., Roberts K.H. (1993). Collective mind in organizations: Heedful interrelating on flight decks. Adm. Sci. Q..

[54] Spreitzer G.M., Lam C.F., Quinn R.W., Spreitzer G.M., Cameron K.S. (2012). Human energy in organizations: Implications for POS from six interdisciplinary streams. The Oxford Handbook of Positive Organizational Scholarship.

[55] Anderson M.H. (2008). Social networks and the cognitive motivation to realize network opportunities: A study of managers’ information gathering behaviors. J. Organ. Behav..

